# CAMKII HYPERACTIVATION IN SKELETAL MUSCLES IS A DRIVER AND POTENTIAL THERAPEUTIC TARGET OF SARCOPENIA

**DOI:** 10.1093/geroni/igae098.2818

**Published:** 2024-12-31

**Authors:** Qinchuan Wang, Michael Bene, William Fountain, Giovanni Rosales-Soto, Erick Hernandez-Ochoa, Tae Hwan Chung, Jeremy Walston

**Affiliations:** Johns Hopkins University School of Medicine, Baltimore, Maryland, United States; Johns Hopkins School of Medicine, Baltimore, Maryland, United States; Johns Hopkins University School of Medicine, Baltimore, Maryland, United States; University of Maryland School of Medicine, Baltimore, Maryland, United States; University of Maryland School of Medicine, Baltimore, Maryland, United States; Johns Hopkins University, Baltimore, Maryland, United States; Johns Hopkins University, Baltimore, Maryland, United States

## Abstract

Sarcopenia, the loss of muscle mass and strength, is a major risk factor for physical frailty in older adults. Ca2+/calmodulin-dependent protein kinase II (CaMKII) is activated by Ca2+ and reactive oxygen species in contracting muscles to enhance muscle performance and adaptation to exercise training. We assessed CaMKII activity in the skeletal muscles of young (4-month-old) and old (20-month-old) C57BL/6J mice and found significantly increased CaMKII activity in old mice at rest, suggesting that CaMKII activity is chronically elevated and disconnected from muscle contractile activity in aged muscles. To determine the role of increased CaMKII, we used an adeno-associated viral vector (AAV9) to express a CaMKII inhibitor, CN19o, in the tibialis anterior (TA) muscles of 20-month-old mice and found a significant improvement of muscle contractile force at 24 to 26 months of age. Conversely, when we expressed a constitutively active CaMKII (CaMKIICA) in the TA muscles of young mice (3-month-old), we observed a striking reduction of muscle contractility. Electromyography studies showed that the muscles expressing CaMKIICA had a subtle but significant reduction of compound muscle action potential (CMAP) that could not explain the reduction of contractile force. Single muscle fiber analyses showed that CaMKIICA did not affect action potential-evoked Ca2+ release. Ongoing research is assessing the clinical relevance of CaMKII activation in humans and examining the histological and transcriptomic profiles of muscles with either inhibited or hyperactivated CaMKII to uncover the mechanisms underlying CaMKII-induced muscle dysfunction. Our findings suggest that CaMKII hyperactivation is a driver and potential therapeutic target of sarcopenia.

